# CD133 expression is correlated with lymph node metastasis and vascular endothelial growth factor-C expression in pancreatic cancer

**DOI:** 10.1038/sj.bjc.6604307

**Published:** 2008-03-18

**Authors:** S Maeda, H Shinchi, H Kurahara, Y Mataki, K Maemura, M Sato, S Natsugoe, T Aikou, S Takao

**Affiliations:** 1 Department of Surgical Oncology and Digestive Surgery, Field of Oncology, Course of Advanced Therapeutics, Kagoshima University Graduate School of Medical and Dental Science, 8-35-1 Sakuragaoka, Kagoshima 890-8520, Japan; 2 Frontier Science Research Center, Kagoshima University, Faculty of Medicine, 8-35-1 Sakuragaoka, Kagoshima 890-8520, Japan

**Keywords:** pancreatic cancer, cancer stem cell, CD133, lymph node metastasis, VEGF-C, predicting factor

## Abstract

Although CD133 has been shown to be a marker for cancer stem cells in various tumours, its expression in pancreatic cancer has not yet been clinically reported. In this study, we investigated the relationship between CD133 expression and clinicopathological factors in pancreatic cancer. Pancreatic head carcinoma specimens from 80 patients who underwent surgical resection were immunohistochemically assessed for CD133, vascular endothelial growth factor (VEGF)-C, CXCR4, CD34, Ki-67, and cytokeratin (CK) expressions. Sixty percentage (48/80) of specimens were CD133-positive, with less than 15% cells per specimen expressing the marker. CD133-positive cells were found at the peripheral site of adenocarcinoma glandular structures and were negative for CK. There was a significant correlation between CD133 expression and clinicopathological factors, including histological type, lymphatic invasion, and lymph node metastasis (*P*=0.0215, 0.0023, and 0.0024, respectively). Vascular endothelial growth factor-C expression was also significantly correlated with CD133 expression (*P*=0.0002). Consequently, the 5-year survival rate of CD133-positive patients was significantly lower than that of CD133-negative patients (*P*=0.0002) and multivariate analysis revealed that CD133 expression was an independent prognostic factor (*P*=0.0103). These results suggest that CD133 expression in pancreatic cancer was significantly associated with lymphatic metastasis, VEGF-C expression, and prognosis.

Pancreatic adenocarcinoma patient survival rates are lower than those of other forms of gastrointestinal malignancies (Silverberg and Lubera, 1988). This could be because of the propensity for early metastasis to regional lymph nodes and the liver, as the presence or absence of lymph node metastasis is an important prognostic factor for patients with pancreatic cancer (Maeda *et al*, 2007).

The existence of cancer stem cells (CSCs) was first demonstrated by transplantation of a small population of harvested leukaemia cells from patients into immunedeficient mice, which then developed the same cancer (Bonnet and Dick, 1997). Since then, recent evidence suggests that a subset of cells within a tumour has stem-like characteristics, including the ability to initiate tumours, high proliferative rates, a high capacity of self-renewal, and the propensity to differentiate into actively proliferating tumour cells (Reya *et al*, 2001; Presnell *et al*, 2002; Pardal *et al*, 2003). These stem-like tumour cells are often associated with elevated expression of the stem cell surface marker CD133 (Neuzil *et al*, 2007).

CD133 (AC133) is a highly conserved antigen and the human homologue of mouse Prominin-1, which was originally identified as a 5-transmembrane cell surface glycoprotein expressed in a subpopulation of CD34+ haematopoietic stem and progenitor cells derived from human fetal liver and bone marrow (Miraglia *et al*, 1997; Yin *et al*, 1997). CD133 expression has been detected in several normal tissues including neuroepithelium, embryonic and adult immature epithelia (Weigmann *et al*, 1997; Corbeil *et al*, 2000; Richardson *et al*, 2004; Shmelkov *et al*, 2005). The association between CD133 and CSCs has been documented in haematological malignancies (Horn *et al*, 1999) and since then, CD133 expression has been identified in various types of solid tumours, including brain tumours (Singh *et al*, 2003, Singh *et al*, 2004; Beier *et al*, 2007), prostate cancer (Collins *et al*, 2005), kidney cancer (Florek *et al*, 2005), melanoma (Klein *et al*, 2007; Monzani *et al*, 2007), ovarian cancer (Ferrandina *et al*, 2007), hepatocellular carcinoma (Suetsugu *et al*, 2006; Ma *et al*, 2007; Yin *et al*, 2007), and colon cancer (Ieta *et al*, 2007; O’Brien *et al*, 2007; Ricci-Vitiani *et al*, 2007).

Transplantation experiments have clearly shown that the behaviour of CD133-positive cancer cells differs from that of CD133-negative cancer cells. For example, transplantation of as few as 100 CD133^+^ glioblastoma cells successfully induced tumours in immunodeficient mice, whereas transplantation of 1 × 10^5^ CD133^−^ cells isolated from the same tumour failed to do so (Singh *et al*, 2004). Moreover, CD133^+^ and CD133^−^ glioblastoma-derived cells showed differential growth characteristics and molecular profiles (Beier *et al*, 2007). CD133^+^ cancer cells of glioma exhibited resistance to radiation (Bao *et al*, 2006a). In prostate cancer, a CSC population with the CD44^+^/integrin*α*2*β*1^hi^/CD133^+^ phenotype has been identified that exhibits extensive proliferation, self-renewal, differentiation, and invasion (Collins *et al*, 2005). In colon cancer, a small number of CD133^+^ cells can maintain themselves as well as differentiate and re-establish tumour heterogeneity after serial transplantation (O'Brien *et al*, 2007; Ricci-Vitiani *et al*, 2007). CD133^+^ cells in colon cancer cell lines have a high degree of tumorigenic ability *in vivo*, and their levels of proliferation, colony formation, and invasive ability were found to be higher than those of CD133^−^ cells *in vitro* (Ieta *et al*, 2007). There are also small populations of CD133^+^ cells in human hepatocellular carcinoma (HCC) cell lines and primary HCC tissues (Yin *et al*, 2007). In contrast with these findings, it was reported that as many as half of Huh-7 cells, a human liver cancer cell line, are CD133^+^ (Suetsugu *et al*, 2006). The CD133^+^ cells possess a greater colony-forming efficiency, higher proliferative output, and greater ability to form tumour *in vivo* (Ma *et al*, 2007). Elevated CD133 expression was found in two of five pancreatic carcinoma cell lines (Olempska *et al*, 2007). Furthermore, a recent report demonstrated that a subpopulation of CD133^+^ CXCR4^+^ cells was responsible for tumour metastasis (Hermann *et al*, 2007).

CD133 mRNA expression appears to be an independent prognostic factor for overall survival, as increased levels were detected in the peripheral blood of cancer patients with bone metastasis (Mehra *etal*, 2006) and were shown to predict colon cancer recurrence (Lin *et al*, 2007). Tumorigenic cells display unique surface marker patterns, such as CD44^+^ in head and neck squamous cell carcinoma (Prince *et al*, 2007), epithelial cell adhesion molecule (EPCAM)^high^ CD44^+^ in colon cancer (Dalerba *et al*, 2007), CD44^+^ integrin*α*2*β*1^+^ in prostate cancer cells (Patrawala *et al*, 2007), CD44^+^ CD24^−/low^ epithelial-specific antigen (ESA)^+^ (Al-Hajj *et al*, 2003), CD44^+^ CD24^−/low^ (Ponti *et al*, 2005) in breast cancer associated with distant metastasis (Abraham *et al*, 2005), and CD44^+^ CD24^+^ ESA^+^ in pancreatic cancer (Li *et al*, 2007).

However, the possible clinical significance of CD133 expression in pancreatic cancer has not been investigated using immunohistochemistry, which is considered a difficult technique because of the inability to detect small numbers of putative stem cells (Miki *et al*, 2007). Nevertheless, immunostained cells may reflect the original features of cells in the tissue, so we used this approach in the present study to detect CD133-positive cells in paraffin-embedded specimens from pancreatic cancer patients. CD133-positive sections were also subjected to analysis of vascular endothelial growth factor (VEGF)-C expression. The purpose of this study was (i) to examine the expression of CD133 in surgical specimens of pancreatic head carcinoma by immunohistochemical methods, (ii) to explore possible correlation between CD133 expression and clinicopathological variables, (iii) to correlate expression of CD133 with VEGF-C, and (iv) to determine the prognostic value of CD133 expression.

## MATERIALS AND METHODS

### Patients and specimens

Eighty patients (52 male and 28 female) who underwent surgical treatment at Kagoshima University Hospital, Kagoshima, Japan for invasive ductal adenocarcinoma of the pancreatic head were included in the study. The patient age range was 42–80 years (mean 66.0 years). All patients underwent macroscopically curative resection by total pancreatectomy, pancreaticoduodenectomy, or pylorus-preserving pancreaticoduodenectomy with lymph node dissection. No preoperative chemotherapy or radiotherapy was administered. Cancer tissue specimens were collected from the patients after informed consent had been obtained, in accordance with the institutional guidelines of our hospital. The number of patients with pT1, pT2, pT3, and pT4 tumours was three (3.8%), four (5.0%), 65 (81.3%), and eight (10.0%), respectively.

Resected primary tumours and lymph nodes were histologically examined by haematoxylin and eosin staining using the tumour-node-metastasis classification system (Sobin and Wittekind, 2002). Histologically, all of the tumours were invasive ductal adenocarcinomas (35 well differentiated, 42 moderately differentiated, and three poorly differentiated). Lymphatic and venous invasions were observed in 69 (86.3%) and 61 tumours (76.3%), respectively. Lymph node metastasis was present in 51 tumours (63.8%).

All patients were assessed by radiography, ultrasonography, and computed tomography every 3 months after discharge. New lesions detected by imaging were considered indicative of relapse. The median follow-up period was 20 months (range 6–168 months). During this period, 29 patients experienced recurrence of liver disease (36.3%).

### Immunohistochemistry

Primary lesions were fixed in 10% formaldehyde, embedded in paraffin, and cut into five 3-*μ*m thick sections every 30 *μ*m. Sections were deparaffinised in xylene, rehydrated in a graded series of ethanol, and incubated in 3.0% hydrogen peroxide in methanol for 10 min to block endogenous peroxidase action. Slides were heated at 100°C in a microwave oven in 10 mM sodium citrate (pH 6.0) for 10 min and cooled to room temperature. After incubation in 1% bovine serum albumin for 30 min at room temperature, sections were incubated overnight at 4°C with an anti-CD133 goat polyclonal antibody (Santa Cruz Laboratory, Santa Cruz, CA, USA; diluted 1 : 200 in phosphate-buffered saline (PBS)), anti-VEGF-C goat polyclonal antibody (Santa Cruz Laboratory; diluted 1 : 200 in PBS), anti-CXCR4 mouse monoclonal antibody (R&D Systems, Minneapolis, MN, USA; diluted 1 : 200 in PBS), and anti-CD34 mouse monoclonal antibody (DAKO Corporation, Carpinteria, CA, USA; diluted 1 : 100 in PBS) for immunostaining microvessels.

The 3-*μ*m thick paraffin sections of primary tumours were deparaffinised, treated with the heat-induced antigen retrieval technique, and epithelial pancreatic carcinoma cells were immunostained for 60 min using cytokeratin (CK) AE1/AE3 (20 : 1 ratio of AE1 to AE3, DAKO Corporation; diluted 1 : 200 in PBS), a mouse monoclonal antibody cocktail that reacts with all epithelial cells of normal pancreatic duct and tumour cells (Pasquinelli *et al*, 1995; Kurahara *et al*, 2007), and Ki-67 mouse monoclonal antibody (DAKO Corporation; diluted 1 : 50 in PBS) that reacts with proliferating cells at any phase of the cell cycle (except for G0 phase) (Igarashi *et al*, 1999). Reactions were developed using the avidin–biotin immunoperoxidase technique (ABC method; Hsu *et al*, 1981). Immunoreactivity was visualised using the Vectastain Elite ABC kit and a 3,3′-diaminobenzidine solution (Vector Laboratories Inc., Burlingame, CA, USA). Sections were then briefly counterstained with haematoxylin.

All immunostained slides were inspected by two independent observers (SM and ST), who had no prior knowledge of the clinicopathological findings. Ten fields (inside the tumour and in the area exhibiting tumour invasion) were selected, and expression was evaluated in 1000 tumour cells (100 cells per field) with high-power (× 200) microscopy. Specimens were defined as positive for CD133 expression if there were tumour cells distinctly stained by anti-CD133 antibody. The presence of VEGF-C and CXCR4 immunoreactivity in over 10% of tumour cells was defined as positive expression. Immunostaining for CD34 was assessed to determine microvessel density (MVD) (Weidner *et al*, 1991; Maeda *et al*, 2007; Mohammed *et al*, 2007). Vessels in the five most highly visualised areas (0.785 mm^2^ per field) by CD34 immunostaining were counted under a × 200 light microscope. The tumour MVD was calculated as the mean value of five fields.

### Statistical analyses

Group differences were statistically analysed using the *χ*^2^ test and *t*-test. The Kaplan–Meier method was used to analyse survival, and the log-rank test was used to estimate differences in survival. Prognostic factors were examined using univariate and multivariate analyses (Cox proportional hazards regression model). *P*-values less than 0.05 were considered statistically significant. All statistical analyses were performed using StatView statistical software version 5.0 (SAS Institute Inc., Cary, NC, USA).

## RESULTS

### Expression of CD133 and CK in pancreatic head carcinoma specimens

**Figure 1:**
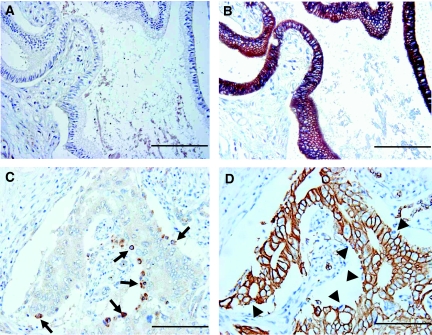
Immunohistochemical staining for CD133 and CK in invasive ductal adenocarcinoma of the pancreas head. (**A**) Normal pancreatic ductal epithelium cells were not stained for CD133. (**B**) The ductal epithelium cells in the consecutive slices of (**A**) were clearly stained by CK antibody. (**C**) CD133 expression appeared to be present at the peripheral portions (facing the interstitial space) of the glandular structures of adenocarcinoma. The percentage of immunoreactive cells (arrows) was estimated to be less than 5% of the tumour cells. (**D**) The CK antibody clearly reacted with the CD133-negative cells of adenocarcinoma, but not with the CD133-positive cells (arrowheads) in the consecutive slices of (**C**). Scale bars, 100 *μ*m.

**Figure 2:**
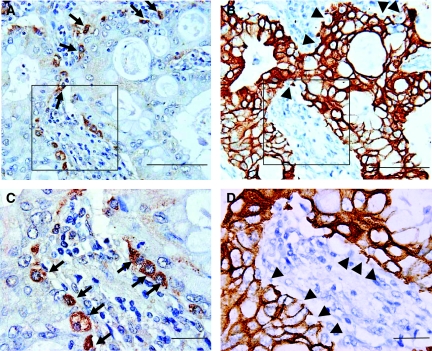
(**A**) CD133 expression (arrows) appeared to be present in the peripheral portions of the glandular structures. The percentage of immunoreactive cells (arrows) was estimated to be more than 5% of the tumour cells. (**B**) The CK antibody clearly reacted with the CD133-negative cells of adenocarcinoma, but not with the CD133-positive cells (arrowheads) in the consecutive slices of (**A**). (**C**) CD133 expression was observed mainly in the cytoplasm of tumour cells (arrows). Shown is a magnified figure described in (**A**) as a box. (**D**) CD133-positive cells were unreactive with the CK antibody (arrowheads) in the consecutive slices of (**C**). Shown is a magnified figure described in (**B**) as a box. Scale bars, 100 *μ*m (**A** and **B**), 20 *μ*m **(C** and **D**).

No CD133 immunoreactivity was observed in normal pancreatic ductal epithelium (Figure 1A), which was clearly stained by the CK antibody (Figure 1B). CD133-positive tumour cells were present in the peripheral site (facing interstitial space) of adenocarcinoma glandular structures in patient samples with both less than (Figure 1C, arrows) and more than 5% CD133-positive tumour cells (Figure 2A, arrows). CD133-negative cells of adenocarcinoma were CK-positive, but CD133-positive cells were not (Figure 1D, arrowheads; Figure 2B and D, arrowheads), which is consistent with previous findings (Hermann *et al*, 2007). Notably, positive CD133 expression was observed in the cytoplasm of carcinoma cells (Figure 2C, arrows).

**Figure 3:**
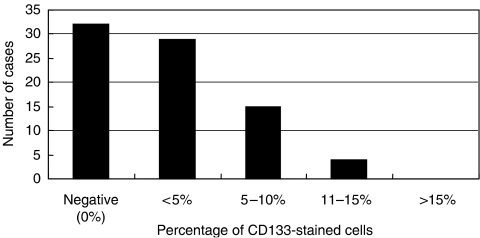
Classification of CD133-positive expression in the invasive ductal adenocarcinoma of the pancreas head samples from 80 patients. Tumour samples were classified into five groups (negative (0%), <5, 5–10, 11–15, and >15%) based on the percentage of stained cells.

CD133 expression was classified into five levels: negative (0%), less than 5, 5–10, 11–15, and more than 15% (Figure 3). Forty-eight (60.0%) of 80 specimens were CD133-positive and the percentage of CD133-positive cells per specimen was less than 15% (Figure 3).

### Correlation between CD133 expression and clinicopathological factors

As shown in Table 1, CD133 expression was significantly correlated with clinicopathological parameters, including histological type (*P*=0.0215), lymphatic invasion (*P*=0.0023), and lymph node metastasis (*P*=0.0024). However, there was no significant association between CD133 expression and age, gender, tumour depth, cancer stage, venous invasion, or liver metastasis (Table 1).

### Relationship between CD133 and VEGF-C expression

**Figure 4:**
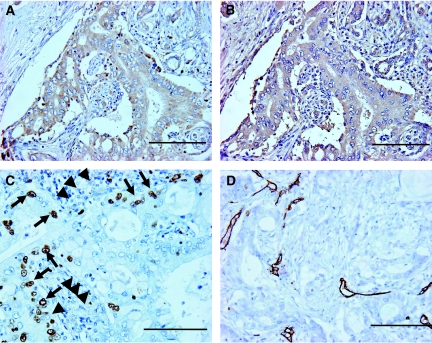
Immunohistochemical staining for VEGF-C, CXCR4, Ki-67, and CD34 in invasive ductal adenocarcinoma of the pancreas head. (**A**) Positive expression of VEGF-C was observed in the cytoplasm of all tumour cells. (**B**) Positive expression of CXCR4 was seen in all epithelial cells of adenocarcinoma. (**C**) Ki-67-positive expression was observed in the nuclei of certain CD133-positive cells (arrows). Some CD133-positive cells were negative for staining with Ki-67 (arrowheads). (**D**) Expression of CD34 was detected in microvessels. The sections shown in (**A**) and (**B**) are the consecutive slices of Figure 1C, and those shown in (**C**) and (**D**) are the consecutive slices of Figure 2A. Scale bars, 100 *μ*m.

When we correlated expression of CD133 with VEGF-C in pancreatic cancers, we observed a significant association between CD133 expression and VEGF-C expression (*P*=0.0002; Table 2). In Figure 4A, a typical pattern of VEGF-C-positive expression is shown as an example in the consecutive slices of Figure 1C.

### Relationship between CD133 and CXCR4 expression

We next examined the correlation between CD133 and CXCR4 expression as it was recently demonstrated that CD133^+^ CXCR4^+^ cells are involved in tumour metastasis (Hermann *et al*, 2007). Eighty-five percentage (68 out of 80) of specimens tested exhibited positive expression for CXCR4. Notably, positive expression of CXCR4 was observed in all epithelial cells of adenocarcinoma. In Figure 4B, a typical pattern of CXCR4-positive expression is shown as an example in the consecutive slices of Figure 1C. The collective data indicate that CXCR4 expression was not associated with CD133 expression (*P*=0.6091; Table 2). Furthermore, in the CD133-positive group, there was no significant association between CXCR4 expression and lymph node metastasis (*P*=0.4425) or lymphatic invasion (*P*=0.5182).

### Relationship between CD133 and Ki-67 expression

When the consecutive slices (of Figure 2A) were reacted with Ki-67 antibody demonstrating proliferating cells, some CD133-positive cells were reactive with Ki-67 antibody (Figure 4C, arrows), but other cells were unreactive (Figure 4C, arrowheads).

### Relationship between CD133 expression and MVD

**Figure 5:**
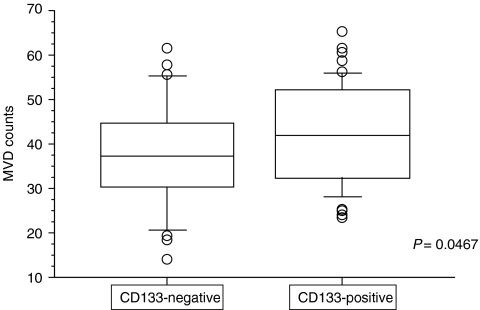
Microvessel density counts after observation of anti-CD34-stained samples (0.785 mm^2^ per field) under a microscope with × 200 power. The MVD (mean±s.d.) of the CD133-positive specimens was significantly different from that of the CD133-negative specimens (43.64±11.26 *vs* 38.18±11.71; *P*=0.0467).

We next correlated expression of CD133 with density of microvessels in pancreatic cancers. Microvessels were delineated by staining with the CD34 antibody. In Figure 4D, tumour with MVD is shown as an example. Notably, MVD was significantly (*P*=0.0467) higher in CD133-positive tumours (mean±s.d.=43.64±11.26) than in CD133-negative tumours (38.18±11.71; Figure 5).

### Prognostic impact of CD133 expression

**Figure 6:**
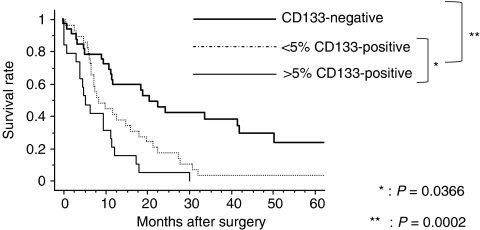
Kaplan–Meier survival curves for patients with more than 5% CD133-positive tumours, less than 5% CD133-positive tumours, and CD133-negative tumours in the pancreatic head carcinomas. There was a significant difference in the 5-year survival rate between patients with tumours that were positive and negative for CD133 expression (*P*=0.0002), and between patients with more and less than 5% CD133-positive tumours (*P*=0.0366).

We examined a possible correlation between patient prognosis and the percentage of CD133-positive cells. The 5-year survival rate of patients with CD133-negative tumours was 23.5%, that of patients with less than 5% CD133-positive cells was 3.4%, and that of patients with more than 5% CD133-positive cells was 0.0%. This finding produced a significant difference in the 5-year survival rate between patients with tumours positive or negative for CD133 expression (*P*=0.0002; Figure 6), and between patients with more or less than 5% cells expressing CD133 (*P*=0.0366; Figure 6).

**Figure 7:**
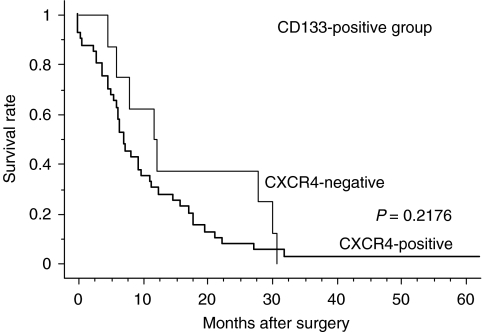
Kaplan–Meier survival curves for patients with CXCR4-positive and CXCR4-negative tumours in the CD133-positive group. There was no significant difference between the patients with tumours that were positive and negative for CXCR4 expression in the CD133-positive group (*P*=0.2176).

In the CD133-positive group, there was no significant difference in the 5-year survival rate between those patients who were positive or negative for CXCR4 expression (*P*=0.2176; Figure 7).

### Patient prognosis

Tables 3 and 4 show the results of univariate and multivariate analyses relating to patient prognosis. Univariate analysis demonstrated that postoperative survival was significantly related to lymphatic invasion, lymph node metastasis, tumour depth, cancer stage, and CD133 expression (*P*<0.05). Multivariate regression analysis found CD133 expression to be an independent prognostic factor, but lymph node metastasis and tumour depth were not.

## DISCUSSION

Recently, it was hypothesised that tumours comprise heterogeneous populations of cells that differ in their abilities to proliferate and exhibit self-renewal. Only a small number of cells, so-called CSCs, can proliferate and exhibit extensive self-renewal; most tumour cells have a limited ability to do so and tend to differentiate into cells that form the tumour mass (Reya *et al*, 2001; Presnell *et al*, 2002; Pardal *et al*, 2003). Cancer stem cells appear to be able to initiate and drive tumour growth in different haematological and solid tumours. CD133, a recently reported prospective marker for CSC, is expressed in a variety of tumours. However, to our knowledge, no attempt has been made to detect CD133 expression in pancreatic cancer specimens.

In this study, we used immunohistochemical staining to detect positive expression of CD133 in 60% of 80 pancreatic head carcinoma specimens, and found that the percentage of CD133-positive cells per specimen was less than 15%. These data are in agreement with the finding that CSCs represent only a very small portion of the total tumour cell population. For example, CD133-positive cells were detected in 0.7–6.1% (Ricci-Vitiani *et al*, 2007), 1.8–24.5% (O'Brien *et al*, 2007), 0.3–3% (Todaro *et al*, 2007) of primary colon cancer cells, and in 0.7–3.2% of primary pancreatic cancer cells using flow cytometric analysis (Hermann *et al*, 2007). Immunohistochemical staining revealed CD133 expression in 1–3% of hepatocellular carcinoma specimens (Ma *et al*, 2007). Notably, we observed no staining for CD133 in normal pancreatic ductal epithelium in the present study (Figure 1A). The present immunohistochemical data appear to support the hypothesis that CD133-positive pancreatic carcinoma cells are involved in the tumorigenic process.

We found that CD133 expression was significantly associated with histological type, lymphatic invasion, and lymph node metastasis (Table 1). It is noteworthy that lymph node metastasis and lymphatic invasion are correlated with CD133 expression, as a recent study demonstrated that a melanoma cell line highly enriched with CD133-expressing cells concomitantly expresses lymphoangiogenic markers, vascular endothelial growth factor receptor (VEGFR)-3 and lymphatic vessel endothelial hyaluronan receptor (LYVE)-1 (Monzani *et al*, 2007). As possible correlation between VEGF-C expression and lymphangiogenesis has previously been described (Kurahara *et al*, 2004; Mohammed *et al*, 2007), we examined VEGF-C expression in pancreatic cancer specimens and found it to be significantly associated with CD133 expression (Table 2). This suggests that CD133-positive pancreatic carcinoma cells promote lymphatic metastasis not only by inducing lymphangiogenesis, but also by facilitating self-entry into the lymphatic system. In view of the potential link to lymphangiogenesis, further studies are needed to address the possible role of CD133 in lymph node metastasis of pancreatic cancers.

Recently, the niche microenvironments of CSCs in brain tumours have been shown to be important for maintaining and self-renewing CSCs through close interaction with endothelial cells (Calabrese *et al*, 2007). In this study, we could not reveal the histological site of such niches, although we observed that CD133-positive cells were localised in the peripheral site (facing interstitial space) of adenocarcinoma glandular structures, and did not express the epithelial differentiation marker CK (Hermann *et al*, 2007). Considering the relationship between CD133-positive cells and lymph node metastasis, lymphangiogenesis and/or lymphatic vessels might be involved in forming the niche microenvironments of CSCs for pancreatic cancer. An understanding of niche histological sites would therefore be of use in preventing progression of pancreatic cancer cells.

High-grade MVD was more frequently found in CD133-positive tumours (Figure 5), reflecting a possible effect of CD133 on tumour vascularity in pancreatic cancer. Bao *et al* (2006b) made similar observations that CD133-positive tumour cells were more vascular than CD133-negative ones. In this context, investigations are currently underway to examine whether CD133-positive cells established from primary pancreatic carcinomas express VEGF more strongly than CD133-negative cells. There is a clear difference between CD133-positive and CD133-negative cells derived from pancreatic cancer regarding angiogenic potential.

A specific subpopulation of CD133^+^ CXCR4^+^ CSCs was recently identified as being responsible for tumour metastasis (Hermann *et al*, 2007). The CXCR4 protein has multiple essential functions, including homing of stem cells and metastasis of cancer cells (Miki *et al*, 2007). Several cancers express CXCR4, and a relationship between CXCR4 expression and malignant potentiality has been suggested (Muller *et al*, 2001; Kaifi *et al*, 2005). In the present study, 85% of specimens exhibited positive expression of CXCR4. Notably, positive expression of CXCR4 was observed in all epithelial cells of adenocarcinoma, which is consistent with previous observations (Gockel *et al*, 2006). Furthermore, there was no significant association between CXCR4 expression and lymph node metastasis, lymphatic invasion, together with prognosis in the CD133-positive group. Taking these findings into consideration, it appears that CXCR4 expression is not associated with CD133 expression, nor with other cancer-related events in the CD133-positive group.

It remains to be determined whether CD133-positive cancer cells exhibit higher degrees of proliferative activity than CD133-negative ones. As a preliminary test, we assessed this possibility using Ki-67 antibody staining and observed the presence of both highly proliferative activity and less proliferative activity of CD133-positive cells. To confirm these findings, we are currently examining the MIB-1 labelling index in CD133-positive and CD133-negative cells.

In conclusion, our immunohistochemical results indicate that CD133 expression in pancreatic head cancer is associated with histological type, lymphatic invasion, lymph node metastasis, VEGF-C expression, and prognosis. Multivariate survival analysis revealed that CD133 expression is an independent prognostic factor. These results suggest that CD133 expression is a useful marker for predicting the outcome of patients with pancreatic cancer. This is the first report of an association between CD133 expression and pancreatic cancer. Understanding the biological function of CD133 expression as a CSC marker in pancreatic cancer will be helpful in elucidating its role in the pathogenesis of pancreatic cancer and developing more effective therapeutic approaches.

## Figures and Tables

**Table 1:** Correlation between CD133 expression and clinicopathologic factors in pancreatic head carcinoma

		**CD133 expression**		
		**Positive**	**Negative**	
	**Total**	***n*=48**	***n*=32**	
	***n*=80 (%)**	**60.0%**	**40.0%**	***P*-value**
*Age (years)*				
Mean±s.d.		65.5±9.9	66.8±8.8	0.6908
				
*Gender*				
Male	52 (65.0)	34 (70.8)	18 (56.3)	0.1803
Female	28 (35.0)	14 (29.2)	14 (43.8)	
				
*Histology*				
Well	35 (43.8)	15 (31.3)	20 (62.5)	0.0215
Moderately	42 (52.5)	31 (64.6)	11 (34.4)	
Poor	3 (3.8)	2 (4.2)	1 (3.1)	
				
*pT*				
pT1	3 (3.8)	1 (2.1)	2 (6.3)	0.3607
pT2	4 (5.0)	1 (2.1)	3 (9.4)	
pT3	65 (81.3)	41 (85.4)	24 (75.0)	
pT4	8 (10.0)	5 (10.4)	3 (9.4)	
				
*pN*				
Negative	29 (36.3)	11 (22.9)	18 (56.3)	0.0024
Positive	51 (63.8)	37 (77.1)	14 (43.8)	
				
*Liver metastasis*				
Negative	51 (63.8)	28 (58.3)	23 (71.9)	0.2171
Positive	29 (36.3)	20 (41.7)	9 (28.1)	
				
*pStage*				
I	6 (7.5)	2 (4.2)	4 (12.5)	0.3540
IIA	22 (27.5)	11 (22.9)	11 (34.4)	
IIB	42 (52.5)	29 (60.4)	13 (40.6)	
III	6 (7.5)	4 (8.3)	2 (6.3)	
IV	4 (5.0)	2 (4.2)	2 (6.3)	
				
*Lymphatic invasion*				
Negative	11 (13.8)	2 (4.2)	9 (28.1)	0.0023
Positive	69 (86.3)	46 (95.8)	23 (71.9)	
				
*Venous invasion*				
Negative	19 (23.8)	9 (18.8)	10 (31.3)	0.1981
Positive	61 (76.3)	39 (81.3)	22 (68.8)	

s.d.=standard deviation.

**Table 2:** Correlation between expression of CD133 and expression of VEGF-C and CXCR4 in pancreatic head carcinoma

	**CD133 expression**		
	**Positive expression *n*=48 (%)**	**Negative expression *n*=32 (%)**	***P*-value**
*VEGF-C expression*			
Negative (*n*=35)	13 (27.1)	22 (68.8)	0.0002
Positive (*n*=45)	35 (72.9)	10 (31.3)	
			
*CXCR4 expression*			
Negative (*n*=12)	8 (16.7)	4 (12.5)	0.6091
Positive (*n*=68)	40 (83.3)	28 (87.5)	

VEGF-C=vascular endothelial growth factor-C.

**Table 3:** Univariate analysis of prognostic factors in pancreatic head carcinoma

**Variables**	***n***	**5-year survival rate (%)**	***P*-value**
*Age (years)*			
Over 65	47	10.4	0.4232
Under 64	33	9.6	
			
*Gender*			
Male	52	10.6	0.9762
Female	28	0.0	
			
*pT*			
pT1, 2	7	42.9	0.0417
pT3, 4	73	5.9	
			
*pN*			
Negative	29	20.4	0.0056
Positive	51	3.2	
			
*Liver metastasis*			
Negative	51	14.2	0.0797
Positive	29	3.4	
			
*pStage*			
I, II	70	11.6	0.0073
III, IV	10	0.0	
			
*Lymphatic invasion*			
Negative	11	30.7	0.0334
Positive	69	6.9	
			
*Venous invasion*			
Negative	19	15.8	0.4944
Positive	61	7.3	
			
*CD133 expression*			
Negative	32	23.5	0.0002
Positive	48	2.1	

**Table 4:** Multivariate analyses of prognostic factors in pancreatic head carcinoma

**Independent factors**	**Univariate *P***	**Multivariate *P***	**Hazard ratio**	**95% confidence interval**
*pT*				
pT1, 2/pT3, 4	0.0417	0.5701	1.355	0.475–3.823
				
*pN*				
Negative/positive	0.0056	0.2141	1.434	0.812–2.534
				
*CD133 expression*				
Negative/positive	0.0002	0.0090	2.151	1.211–3.869
